# Prudential Use of the Morally Controversial COVID-19 Vaccines

**DOI:** 10.1177/00243639211017697

**Published:** 2021-05-25

**Authors:** Nicanor Pier Giorgio Austriaco

**Affiliations:** 1 Providence College, Providence, USA and University of Santo Tomas, Manila, Philippines

**Keywords:** Abortion, COVID-19 vaccines, Fetal cell lines, HEK293, Prudence

## Abstract

In recent months, there has been a lot of debate surrounding the use of those COVID-19 vaccines that have been either tested or manufactured with cell lines that were isolated from the remains of an aborted fetal child. Most faithful and orthodox Catholic moral theologians, among whom I count myself, have concluded that their use is not intrinsically evil. Therefore, like every other decision that falls into the category of actions that are not intrinsically evil, the decision to be vaccinated with these morally controversial vaccines has to be governed by the virtue of prudence. It is a decision that calls for a wisdom that properly sees this action within the constellation of actions that propels the human agent to the heights of holiness. This is why prayer is so essential for authentic moral judgment. With prayer, we ask the Holy Spirit who is the all-prudent one to guide our actions so that we can choose and act well not only for our only well-being but for the well-being of all. Acts that are not themselves intrinsically evil are deemed virtuous or not within the narrative of the individual person’s life.

From the perspective of the natural law, there are two kinds of human acts. On the one hand, there are those actions that are inherently incompatible with human flourishing.^1^ They can never be reconciled with an authentic account of the human good and are therefore evil at all times and in all places. In the Catholic moral tradition, these acts are called intrinsically evil acts. Examples of these acts include the acts that we call abortion, euthanasia, slavery, and theft. Each of these actions is specified by a distinctive moral object that makes the action what it is. An individual seeking to grow in virtue and in holiness would never choose to willingly perform these actions under any set of circumstances.

On the other hand, there are those acts that can be made compatible with human flourishing depending upon the particular circumstances that surround the act. Most of the daily actions that we perform in our ordinary lives fall into this category. Take the action we call eating a meal. A person could eat a healthy, low-salt lunch, or she could choose to eat a high-sodium meal. For most persons, the former would be a virtuous act, while the latter would not because the first meal would promote the good of human health while the latter would not. However, I know of a priest and his sister with hyponatremia, that is, with low levels of blood sodium, who were told by their primary care physicians that a high-sodium meal may actually be beneficial for their health and well-being. In their particular circumstances, eating a high-sodium meal would be virtuous and skipping out on the salt would not be. In these cases of acts that are not intrinsically evil, whether an action is judged to be virtuous or not depends on the particular narrative story of the human agent.

As the classical ethical tradition sees it, every individual needs the virtue of prudence to judge whether or not to perform an action that falls into the second category of acts described above. St. Thomas Aquinas defined prudence as wisdom concerning human affairs (*STh* II-II.47.2 ad 1) or right reason with respect to action (*STh* II-II.47.4). It is an intellectual knowing that enables us to see whether our actions would or would not allow us to fulfill our proper end (*STh* I-II.57.5). The individual with hyponatremia who sees that eating this high-sodium meal is good for him, even if it is too salty for him, would be prudent. In contrast, the patient who rejects the meal simply because she does not like its flavor would not be.

In recent months, there has been a lot of debate surrounding the use of those COVID-19 vaccines that have been either tested or manufactured with cell lines that were isolated from the remains of an aborted fetal child. At the time of this writing, in the United States, these FDA-approved vaccine brands include Pfizer and Moderna, which were tested with HEK293 cells, and Johnson & Johnson (JNJ), which was manufactured with the per.C6 cell line.

Is the use of these vaccines intrinsically evil? Most faithful and orthodox Catholic moral theologians, among whom I count myself, have concluded that it is not.^2^ I will not repeat our arguments here. This conclusion has been confirmed by the magisterium of the Catholic Church at both the level of the universal Church^3^ and of the local churches teaching through their bishops’ conferences.^4^ Had there been any vaccines whose use would have constituted an intrinsically evil act, the Church would have advised her people to avoid them in every and all circumstances.

Therefore, like every other decision that falls into the category of actions that are not intrinsically evil, the decision to be vaccinated with these morally controversial vaccines has to be governed by the virtue of prudence. It is a decision that calls for a wisdom that properly sees this action within the constellation of actions that propels the human agent to the heights of holiness. Let me illustrate what I mean here by describing three persons who are faced with the decision to vaccinate or not to vaccinate with the JNJ vaccine which is made available to them. The JNJ is one vaccine that was manufactured using a fetal cell line that has its origins in an elective abortion.

In the first case, we have a twenty-six-year-old, single woman who works as a data analyst. She lives by herself, and she has worked from home for most of the pandemic. She decides that she is going to forgo being vaccinated with the JNJ vaccine because she wants to be a prophetic witness to the sanctity of human life. Instead, she will wait until a noncontroversial option is available in her county. She has made a virtuous decision.

In the second case, we have a twenty-six-year-old physical therapist who works for a nursing home where several of her residents were not able to be vaccinated because of their poor health. If she comes to me and tells me that she wants to forgo the JNJ vaccine to be a prophetic witness, I would tell her that I salute her for her decision. However, I would also counsel her that she should also resign from her job at the nursing home: to continue working there would be unjust because it would risk exposing her residents to COVID-19 because she chose not to be vaccinated. Her resigning would perfect her truly heroic and prophetic act.

However, if she tells me that she could not give up her job because she needs the income to support her family, then I would have to tell her that she in fact has a moral duty to be vaccinated once a vaccine is made available to her, even if it were one of the morally controversial vaccine brands. In these specific circumstances, foregoing the vaccination would not be an act of prophetic witness. Regardless of her good intentions, it would be an unjust act that would imperil not only others but also her salvation.

In the third case, we have a twenty-six-year-old pro-life activist who is well-known in her local community for her pro-life advocacy. Like the data analyst in the first scenario, she lives by herself and is able to work from home. She tells me that she too would like to forego the morally controversial vaccines. Again, I would salute her. However, I would also suggest to her that given her work, she, unlike the data analyst, may actually have a moral duty to be a prophetic witness in this matter. Her choosing to receive the JNJ vaccine could risk causing grave scandal in a way that the same decision would not with either of our other twenty-six-year olds. Acts that are not themselves intrinsically evil are deemed virtuous or not within the narrative of the individual human agent’s journey toward holiness.

Finally, I have to emphasize that the circumstances that should guide our moral judgments should not be limited to those circumstances that involve only our individual lives. To be truly prudent and to be truly wise, we need to consider the wider context of our moral judgments as well.

Returning now to the morally controversial vaccines, the emergency use authorization of the JNJ single-shot vaccine triggered a debate among Catholics in the United States over the use of this morally controversial shot. Despite the pronouncements of the Vatican and the US Conference of Catholic Bishops that these vaccines can be taken, some groups were arguing that the use of the morally controversial vaccines was to be avoided at all cost and that using these vaccines constituted a greater evil than foregoing them to bear prophetic witness. No one considered the global implications of this rhetoric in their analysis.

I am a Filipino-American molecular biologist who is helping to manage the COVID-19 pandemic in the Philippines. I have also been involved in the procurement and deployment of our vaccine portfolio. Where American Catholics may have a choice of vaccines, their Filipino counterparts have none. As I write this in the middle of March, the United States has administered 100 million doses of the COVID-19 vaccines in a population of 330 million citizens while the Philippines has only vaccinated 110 thousand of its 112 million citizens with their first dose. The stark contrast in vaccination numbers is driven by a severe global shortage of vaccines that has arisen in part because of vaccine hoarding: the United States has secured 3,360 million doses of vaccines for its 330 million citizens—that is ten doses for every American!—while the Philippines has only been able to secure 110 million doses for its 110 million citizens—that is one dose for every Filipino, when most vaccines require two doses per person.^5^

Moreover, the Philippines will receive its vaccines in spurts over the course of the year. As a result, Filipinos will have to be vaccinated with whichever vaccine is available when they reach the front of the vaccination line. There is no option for choice. There is no option for waiting because there will be no surplus vaccines at the end of the vaccination schedule. Vaccine choice in the United States, where it exists, is a privilege facilitated by the grave injustice of vaccine hoarding.

The Philippines is pro-life by constitutional statute and profoundly Catholic. When American Catholics speak about the lesser evil of using the morally controversial vaccines, they put millions of Filipinos in an ethical dilemma. No one wants to participate in any evil so rhetoric that categorizes vaccine use as a “lesser evil” triggers vaccine hesitancy in my homeland. Moreover, as we have seen, this rhetoric is unnecessary. We can all use the language of virtue to reveal the appropriate parameters for this moral discourse rather than the language of evil. And we can acknowledge that different persons legitimately reach different conclusions because of differing circumstances. This would be the prudential approach.

In sum, most moral decision making in everyday life is not governed only by laws and commandments that rule out and rule in specific acts. Most moral decision making—including moral decision making in the clinical setting—demands the virtue of prudence that is able to discern which of any number of actions is the one that best will make us into saints in a particular time and place. This is why prayer is so essential for authentic moral judgment. With prayer, we ask the Holy Spirit who is the all-prudent one to guide our actions so that we can choose and act well not only for our only well-being but for the well-being of all.

## Notes

1. John Paul II, Veritatis Splendor, §§79-83.

2. Nicanor Pier Giorgio Austriaco, OP, “Moral Guidance on Using COVID-19 Vaccines Developed with Human Fetal Cell Lines,” *Public Discourse*, May 26, 2020. Available at https://www.thepublicdiscourse.com/2020/05/63752/. Also see Christopher O. Tollefsen, “Research Using Cells of Illicit Origin and Vaccines from Fetal Tissue,” *Public Discourse*, May 27, 2020. Available at https://www.thepublicdiscourse.com/2020/05/63447/; and “Statement from Pro-Life Catholic Scholars on the Moral Acceptability of Receiving COVID-19 Vaccines,” *Public Discourse*, March 11, 2021. Available at https://www.thepublicdiscourse.com/2021/03/74594/

3. Congregation for the Doctrine of the Faith, “Note on the Morality of Using Some Anti-Covid-19 Vaccines,” December 21, 2020. Available at https://www.vatican.va/roman_curia/congregations/cfaith/documents/rc_con_cfaith_doc_20201221_nota-vaccini-anticovid_en.html and Vatican COVID-19 Commission in collaboration with the Pontifical Academy for Life, “Note of the Vatican Covid-19 Commission in collaboration with the Pontifical Academy for Life ‘Vaccine for all. 20 points for a fairer and healthier world’,” December 29, 2020. Available at https://press.vatican.va/content/salastampa/en/bollettino/pubblico/2020/12/29/201229c.html.

4. US Conference of Catholic Bishops, “Moral Considerations Regarding the New COVID-19 Vaccines,” December 11, 2020. Available at https://www.usccb.org/moral-considerations-covid-vaccines.

5. https://www.unicef.org/supply/covid-19-vaccine-market-dashboard.

